# Current Opportunities for Clinical Monitoring of Axonal Pathology in Traumatic Brain Injury

**DOI:** 10.3389/fneur.2017.00599

**Published:** 2017-11-20

**Authors:** Parmenion P. Tsitsopoulos, Sami Abu Hamdeh, Niklas Marklund

**Affiliations:** ^1^Section of Neurosurgery, Department of Neuroscience, Uppsala University, Uppsala, Sweden; ^2^Hippokratio General Hospital, Aristotle University, Thessaloniki, Greece; ^3^Department of Clinical Sciences Lund, Neurosurgery, Skåne University Hospital, Lund University, Lund, Sweden

**Keywords:** traumatic brain injury, diffuse axonal injury, monitoring, neurocritical care, neuroimaging, biomarkers, microdialysis

## Abstract

Traumatic brain injury (TBI) is a multidimensional and highly complex disease commonly resulting in widespread injury to axons, due to rapid inertial acceleration/deceleration forces transmitted to the brain during impact. Axonal injury leads to brain network dysfunction, significantly contributing to cognitive and functional impairments frequently observed in TBI survivors. Diffuse axonal injury (DAI) is a clinical entity suggested by impaired level of consciousness and coma on clinical examination and characterized by widespread injury to the hemispheric white matter tracts, the corpus callosum and the brain stem. The clinical course of DAI is commonly unpredictable and it remains a challenging entity with limited therapeutic options, to date. Although axonal integrity may be disrupted at impact, the majority of axonal pathology evolves over time, resulting from delayed activation of complex intracellular biochemical cascades. Activation of these secondary biochemical pathways may lead to axonal transection, named secondary axotomy, and be responsible for the clinical decline of DAI patients. Advances in the neurocritical care of TBI patients have been achieved by refinements in multimodality monitoring for prevention and early detection of secondary injury factors, which can be applied also to DAI. There is an emerging role for biomarkers in blood, cerebrospinal fluid, and interstitial fluid using microdialysis in the evaluation of axonal injury in TBI. These biomarker studies have assessed various axonal and neuroglial markers as well as inflammatory mediators, such as cytokines and chemokines. Moreover, modern neuroimaging can detect subtle or overt DAI/white matter changes in diffuse TBI patients across all injury severities using magnetic resonance spectroscopy, diffusion tensor imaging, and positron emission tomography. Importantly, serial neuroimaging studies provide evidence for evolving axonal injury. Since axonal injury may be a key risk factor for neurodegeneration and dementias at long-term following TBI, the secondary injury processes may require prolonged monitoring. The aim of the present review is to summarize the clinical short- and long-term monitoring possibilities of axonal injury in TBI. Increased knowledge of the underlying pathophysiology achieved by advanced clinical monitoring raises hope for the development of novel treatment strategies for axonal injury in TBI.

## Introduction

Traumatic brain injury (TBI) is a significant cause of morbidity and mortality worldwide (1–5). Mortality due to severe TBI can reach 40% with high rates of disability among the survivors (6–8). Cognitive, behavioral, and emotional impairments are common and particularly disabling post-TBI and can persist into the chronic stage (9–12). Widespread injury to the white matter tracts, a key feature of TBI, disrupts neuronal networks and impairs information processing which contributes to the cognitive impairments observed post-TBI (9–11).

Axonal injury was initially described by Strich in 1956, who observed diffuse axonal degeneration at autopsy of severe TBI patients (13). Axonal pathology was later established as a separate TBI entity by Adams and colleagues in 1982 (14). When axonal damage occurs in multiple brain locations in clinical TBI, it is named diffuse axonal injury (DAI) (15–21).

In the preclinical setting, the term traumatic axonal injury has been applied to describe axonal damage, with the term DAI used to express its clinical counterpart (5, 19). Thus, DAI is a clinical entity characterized by radiological and/or histological findings suggestive of axonal pathology at certain predilection sites, particularly the gray/white matter interface, the corpus callosum, and the brain stem (14, 15). DAI remains a challenging clinical entity with a frequently unpredictable course and outcome. To date, there are limited therapeutic options reflecting the incomplete knowledge of the underlying pathophysiology of DAI. However, it has been established that axonal injury results from a highly dynamic process involving a cascade of events that may evolve over time leading to progressive white matter atrophy with variable clinical impact (19, 22–24). Therefore, detection and monitoring of DAI is relevant from the acute to chronic phase, in order to evaluate its severity, seek treatment options and better predict clinical outcome.

In this review, an overview of the current clinical possibilities for monitoring of DAI is provided. A literature search was performed in PubMed, Scopus and ISI Web of Knowledge. Experimental/preclinical studies on axonal injury in TBI were excluded from the overview with the exception of those describing key pathophysiological mechanisms. Articles where the injury type was not mentioned, was unclear or encompassing only focal TBI were also excluded from the analysis. Articles on mild, moderate, and/or severe TBI, DAI, and traumatic axonal injury were extracted, further screened and were included if the investigated mechanisms involved aspects of axonal/white matter injury. The literature on mild TBI, considered a diffuse TBI subtype with features of axonal and white matter pathology, was thus also included in our search.

## Biomechanics and Structural Changes in DAI

**Figure 1:**
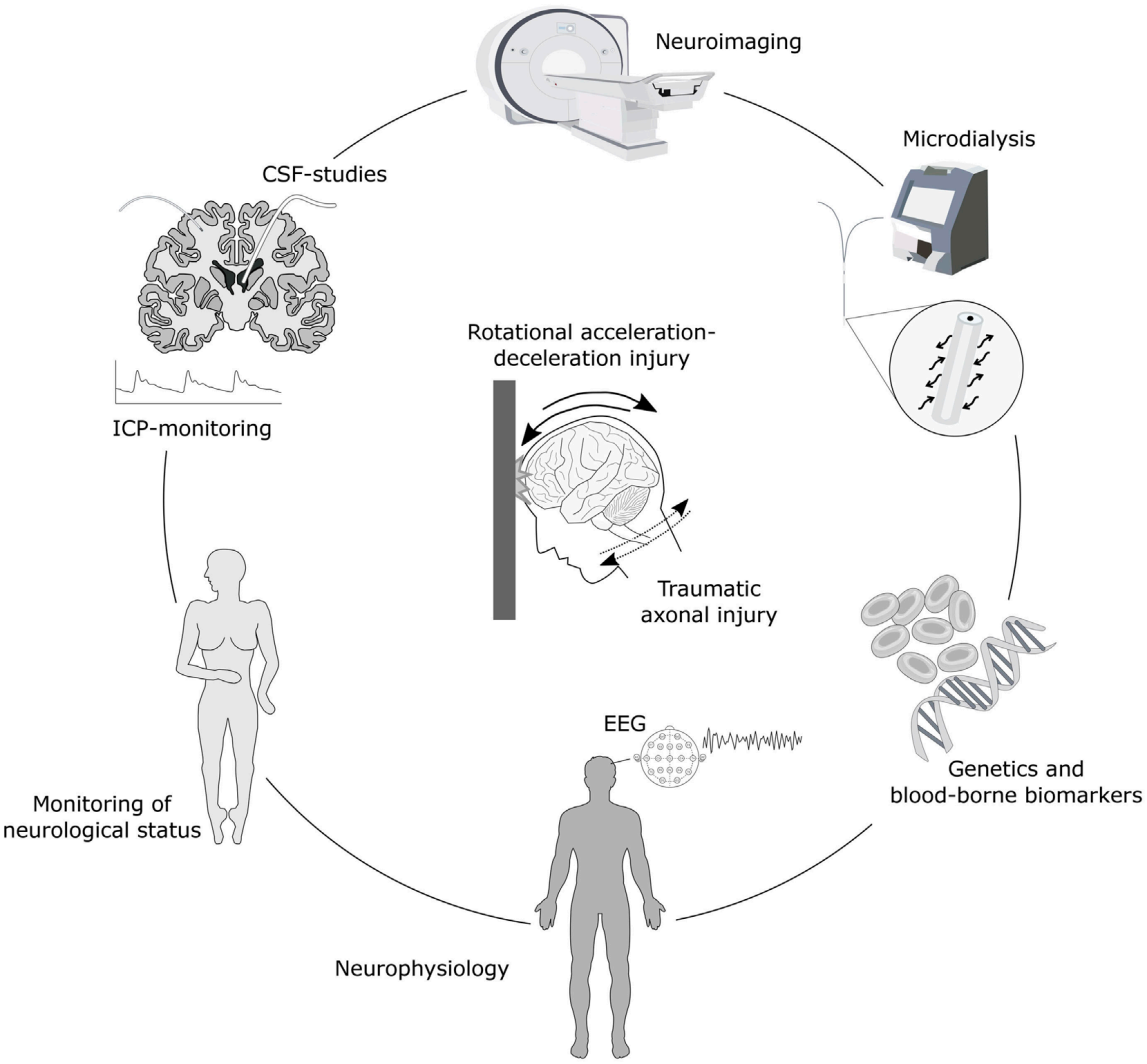
Schematic illustration of monitoring options for axonal injury. Biomechanically, traumatic axonal injury results from head impact with rotational acceleration-deceleration forces. Detection and monitoring of axonal injury is possible with numerous advanced neuroimaging techniques such as magnetic resonance imaging (MRI), including diffusion tensor imaging (DTI) and magnetic resonance spectrometry (MRS), as well as neuromolecular imaging by single-photon emission computed tomography (SPECT) and/or positron emission tomography (PET). Axonal injury also results in the secretion of various biomarkers into the interstitial fluid (ISF), cerebrospinal fluid (CSF) and the bloodstream which can be detected in ISF using microdialysis, in CSF by sampling through an external ventricular drainage or through lumbar puncture, and in serum by blood sampling. These biomarkers provide clues of temporal patterns of axonal injury and ongoing secondary injury processes and may be associated with outcome. Monitoring of axonal injury progression may also be achieved by placement of an intracranial pressure (ICP) monitoring device for continuous surveillance of ICP, neurophysiological methods such as electroencephalography (EEG) and periodic assessments of neurological status including level of consciousness. Furthermore, the genetic profile may add additional information of risk for secondary injury cascades and neurodegenerative development.

The predominant mechanism in the development of axonal injury in TBI is mechanical shearing and stretch forces produced by inertial acceleration/deceleration stresses to the head (25–28) (Figure 1). This inertial loading triggers dynamic shear, tensile, and compressive strains within the brain. Consequently, certain parts of the brain move at a slower pace relative to others, leading to deformation of the brain tissue (17, 29). In the preclinical TBI setting, unmyelinated axons sustained more injury compared to myelinated ones, suggesting that axons are unequally vulnerable (30).

Under normal conditions, brain tissue can withstand stretches and easily return to its original geometry without any resulting injury. In contrast, when the strain is rapidly applied, the brain tissue loses its plasticity and acts stiffer, becoming more vulnerable and brittle (31). In particular, axons in the white matter appear poorly prepared to withstand injury from rapid mechanical brain deformation at time of TBI (17), resulting in injury to the axonal cytoskeleton (17, 32, 33). Nevertheless, the development and severity of axonal injury is dependent on both the magnitude and rate of strain during impact (17).

Primary axotomy in humans is rare with secondary (delayed) axotomy being the most likely mechanism leading to axonal disconnection (5, 19, 34, 35). Experimental evidence indicates that mitochondrial dysfunction (5, 36), as well as TBI-induced inflammatory responses, contribute to the secondary axonal injury (5, 37, 38). In addition, impaired axonal transport causes axonal swelling over time post-injury, leading to the accumulation of numerous potential biomarkers which may then be released into the surrounding tissue (see later section of this review).

## Clinical Features and Neurocritical Care Monitoring of Axonal Injury

### Clinical Characteristics

From a clinical point of view, initial loss of consciousness and coma as well as later features such as prolonged vegetative state or cognitive impairment can be characteristics of both focal TBI and DAI, although may be more frequently observed in the latter (29, 39). The presence of a decreased level of consciousness and coma is commonly a result of axonal injury in the diencephalon and/or the brain stem (29, 40, 41). In DAI survivors, cognitive dysfunction, mood disorders, and behavioral problems are frequent and result in a decreased quality of life (17, 29, 42). In particular, memory impairment and problems in executive functioning are frequent in DAI (43, 44), and so is impaired information–processing speed (45). In addition, motor weakness may be caused by injury to the pyramidal tract (46), which is more frequently encountered in DAI compared to focal TBI (29).

By definition, axonal injury is difficult to diagnose using only clinical signs and symptoms. Some clinical features of axonal injury are presumably to a large extent related to the anatomic distribution of DAI. Based on the limited ability of computed tomography (CT) and standard T1- and T2-weighted magnetic resonance imaging (MRI) sequences to precisely detect the underlying axonal injury in TBI, it has been difficult to confirm and diagnose DAI with certainty (17). However, neuropsychology testing after release from hospital can detect cognitive and memory deficits as well as slowed mental processing, characteristics which, when combined with knowledge of the underlying injury mechanisms (Figure 1) and neuroimaging findings, may be highly suggestive of DAI. These features can help confirm the diagnosis of moderate or severe DAI with a relatively high degree of certainty in TBI patients (29, 47, 48).

In addition, advanced neuroimaging has recently enabled improved visualization of surrogate markers for the histopathological features of DAI, and for subsequent surveillance of secondary injury processes. Injury to white matter tracts interconnecting cortical regions, disrupting large scale brain networks of particular importance for complex cognitive functions (49, 50), are now possible to estimate using modalities like diffusion tensor imaging (DTI) and be correlated with cognitive and behavioral deficits observed using neuropsychology testing (10, 50, 51).

### Intracranial Pressure (ICP) Monitoring of DAI

Intracranial pressure monitoring remains a cornerstone in the management of severe TBI patients, although the incidence of raised ICP in DAI is not well established. Maximum ICP has been correlated to the number of identifiable white matter lesions on MRI (52), and a relationship with the Marshall CT classification score and ICP levels was suggested (53, 54). In some studies of severe DAI patients, ICP was not elevated (55), whereas others found increased ICP in most TBI patients (56–58). In an early study of ICP monitoring in TBI patients, of whom 61 had a normal CT scan, no ICP elevations were observed unless the patient was aged >40 years, had unilateral or bilateral motor posturing or episodes of systolic blood pressure <90 mmHg (59). In contrast, another study found elevated ICP despite the absence of mass lesions, midline shift, or compressed basal cisterns on the initial CT scan (60). Later, less ICP elevations were observed in DAI compared to other TBI subtypes and it was suggested that ICP monitoring could be omitted in DAI (55). However, in that particular study, 10% of patients had ICP >20 mmHg, and two patients required treatment for elevated ICP. Similar patterns of transient ICP elevations triggered by neurocritical care events in DAI patients were also observed (61). Recently, ICP was analyzed in MRI-verified DAI patients, and although persistently raised ICP during the first 96 hours of monitoring was not seen, 20% of patients required treatment for transient ICP elevations (62).

Thus, the use of ICP monitoring in DAI is controversial (63). Although it was suggested that individuals with DAI documented by neuroimaging may not require treatment for elevated ICP, high ICP values were still frequently encountered in such patients. The use of ICP monitoring in comatose patients with initial normal CT scan or CT scan with minimal findings has also been questioned and recommended only in the presence of radiological worsening (64). On the other hand, in comatose patients with diffuse TBI with evidence of brain swelling on CT scan, ICP monitoring is indicated in the early post-injury period (64). It should be also noted that DAI patients with effaced basal cisterns on CT scan carry a high risk of increased ICP (58, 65).

In summary, studies evaluating the incidence of elevated ICP in DAI patients are scarce and provide contradictory results. Repeated clinical examinations and neuroimaging may be possible alternatives for monitoring of DAI patients when the initial CT scan is free from or shows only minimal abnormalities, since these patients may have a low risk of intracranial hypertension (63). Nevertheless, although it has not been firmly shown that outcome is improved, ICP monitoring in DAI patients with reduced level of consciousness and pathological findings on CT scan is recommended in the initial post-injury period (64, 66, 67).

### Monitoring of Cerebral Blood Flow (CBF) and Brain Oxygenation

Perfusion CT or xenon-enhanced CT (Xe-CT) are both rapid and widely available techniques for the evaluation of CBF. For Xe-CT, a mobile CT scanner enabling bedside measurement of CBF is used (68). Although clinical experience in DAI is still limited, significant CBF alterations seem less frequent than in focal TBI (69–71). These imaging techniques, however, allow only intermittent CBF measurements and transient CBF impairment in the intervals between examinations cannot be established. Continuous monitoring of CBF is possible using thermal diffusion or laser Doppler methods, both requiring insertion of an intraparenchymal probe to assess focal CBF in a small brain volume (72, 73). Clinical experience with these techniques is still limited, and to date, there are no studies specifically evaluating local CBF measurements in DAI.

Cerebral blood flow may also be indirectly estimated using jugular venous oxygen saturation (Sjvo_2_) and brain tissue oxygenation (PBto_2_). Sjvo_2_ can be measured using a fiberoptic probe placed in the jugular bulb and ranges between 55–75% under normal conditions. Low Sjvo_2_ values may suggest hypoperfusion and ischemia and episodes of desaturation correlate with poor outcome (74). On other hand, high values >75% may represent hyperemia and also correlate with brain infarctions, since oxygen is not extracted from irreversibly injured brain tissue.

PBto_2_ measurements require a sensor to be inserted in deep white matter, and allow regional measurements of cerebral oxygenation. In the uninjured brain, PBto_2_ values are >20 mmHg while critical hypoxia may develop with values <10 mmHg. Although reductions of PBto_2_ have been associated with poor outcome in TBI (75), and current treatment recommendations suggest interventions when PBto_2_ falls below 15 mmHg (76), no studies have to our knowledge focused on the clinical impact of PBto_2_ in DAI patients.

Available methods for CBF measurements as well as brain oxygenation cannot be firmly recommended in DAI in view of the limited clinical experience with these methods. Nonetheless, they are expected to play a greater role in the future especially in multifocal/mixed cases with elevated ICP and impaired CPP as a complement to ICP-CPP guided treatment protocols.

### Electroencephalography (EEG)

In TBI patients, continuous EEG (cEEG) has been proven useful for the monitoring of seizure activity and the depth of sedation especially in those on barbiturate coma (77, 78). The use of cEEG in TBI is also indicated for the detection and treatment of non-convulsive seizures (NCS), a common risk in severe TBI patients (79, 80). Although only low quality evidence exists, cEEG monitoring may be recommended in TBI patients with unexplained behavioral alterations or sudden changes in mental state and/or altered consciousness, and to rule out NCS especially in penetrating injuries, large intracranial lesions, and depressed skull fractures (79).

There is limited data on the use of EEG/cEEG in the monitoring of DAI. In a study of 90 patients after diffuse TBI, where EEG recording was applied in the early post-injury phase, the EEG patterns correlated with prognosis (81). Specifically, most DAI patients with “benign” EEG patterns (stage 1; normal records with preserved activity, stage 2; reactive with rhythmic theta activity dominant, stage 3; usually reactive spindle coma where sleep patterns of stage 2 demonstrated rhythmic spindles) survived while most patients with “malignant” EEG findings (low amplitude delta activity, burst suppression pattern, alpha pattern coma) died (81). Following blast TBI, typically resulting in a degree of white matter/axonal injury, reduced EEG phase synchrony in the frontal area was associated with axonal injury on DTI (82, 83).

To date, the role of EEG in the monitoring of DAI has not been established. Although there is evidence to support that cEEG monitoring may be useful for the diagnosis of NCS in severe TBI, there is insufficient data in DAI at present. This monitoring modality has also not been shown to improve outcome and/or alter treatment in DAI patients. To date, it should primarily be regarded as a scientific tool awaiting additional studies evaluating its clinical role in the multimodality monitoring of DAI.

## Neuroimaging

In recent years, advances in neuroimaging have facilitated DAI diagnostics as well as allowed for more accurate prognosis and monitoring of ongoing, secondary axonal injury. While image acquisition speed, accessibility and accuracy in detecting traumatic intracranial hemorrhages make CT the leading neuroimaging modality in the acute evaluation of TBI patients, its utility in DAI is limited. Although traumatic edema of the brain and petechial hemorrhages in the white matter indicate DAI, CT is generally insensitive for subtle axonal lesions. Hemorrhagic lesions in deep-seated predilection sites for DAI such as the corpus callosum and the rostral brain stem are rarely seen on CT. In addition, non-hemorrhagic lesions, which have been linked to poor outcome (84, 85), are not detectable (86). As discussed previously, there is a degree of axonal injury caused by the initial impact although most axonal pathology in DAI is a delayed secondary event evolving over days to weeks resulting in clinical deterioration of the patient. Thus, the admission CT in combination with the clinical picture following TBI may indicate DAI, but for the confirmation of diagnosis, monitoring of the progression of axonal injury and adequate prognosis, more advanced imaging modalities are needed.

### Magnetic Resonance Imaging

**Figure 2:**
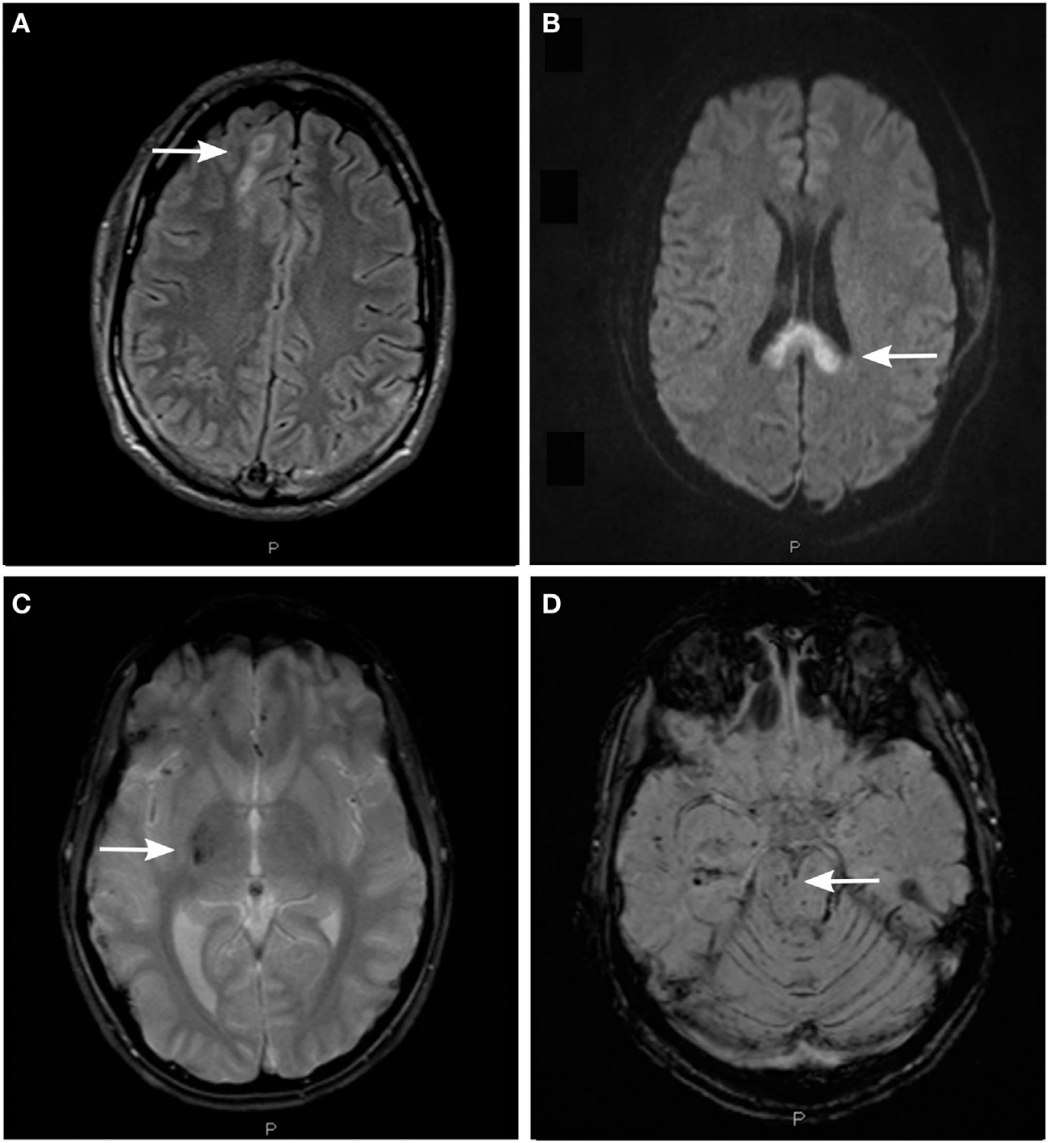
Detection of axonal injury with conventional magnetic resonance imaging (MRI) using different MRI sequences. **(A)** Fluid-attenuated inversion recovery (FLAIR) image depicting non-hemorrhagic diffuse axonal injury (DAI)-associated lesions in the subcortical white matter of the right cerebral hemisphere (arrow). **(B)** Diffusion-weighted image (DWI) depicting non-hemorrhagic DAI-associated lesions in the body and splenium of the corpus callosum. **(C)** T2*-weighted gradient echo (T2*GRE) image depicting hemorrhagic DAI-associated lesions in the right thalamus and putamen (arrow). **(D)** Susceptibility-weighted image (SWI) depicting hemorrhagic DAI-associated lesions in the right mesencephalon (arrow) and in the white matter of right temporal lobe.

Magnetic resonance imaging is a more sensitive modality for visualization of DAI-associated lesions and can detect microscopic amounts of blood, as well as non-hemorrhagic lesions secondary to axonal strain (Figure 2). In addition, MRI provides means to assess and visually reconstruct white matter tracts following DAI, with high sensitivity for lesion detection using DTI. Furthermore, neurochemical alterations following axonal injury can be detected with magnetic resonance spectrometry (MRS).

### Conventional MRI Sequences for DAI Monitoring

Magnetic resonance imaging sequences sensitive to hemorrhagic lesions include T2*-weighted gradient echo (T2*GRE) and susceptibility-weighted imaging (SWI). Both sequences can detect microhemorrhages, taking advantage of the paramagnetic properties of hemoglobin degradation products. The lesions are typically seen as small hypointense foci in DAI predilection sites and appear larger than their true size due to the magnetic field distortion. Hemorrhagic lesions seem to be fairly stable over time although some reduction of lesion numbers may be seen in the chronic phase following DAI (84, 87). Adding sensitivity of microhemorrhage detection in deep-seated brain regions, SWI has emerged as a preferred MRI sequence (88, 89) particularly in brain regions such as central brain stem previously difficult to assess (62). However, this sequence may be more complicated to interpret, since deoxygenated blood in veins can mimic hemorrhagic lesions. Nonetheless, lesions seen on SWI sequence correlate strongly to outcome (62, 90), in contrast to T2*GRE (62, 87, 91).

The Fluid-Attenuated Inversion Recovery (FLAIR) sequence facilitates detection of non-hemorrhagic lesions adjacent to cerebrospinal fluid (CSF) spaces. This sequence is useful for visualizing axonal injuries in periventricular white matter, the corpus callosum and the brain stem (84, 85). However, its capacity to visualize axonal injury is highly dependent on the timing. Lesions demonstrated in the acute phase represent tissue edema, but some seem to disappear already by 3 months post-injury (84). On the other hand, FLAIR lesions represent encephalomalacia (softening or loss of brain tissue) or tissue gliosis at the long-term, chronic phase in DAI (92).

The diffusion-weighted imaging (DWI) sequence is sensitive to the microscopic motion of water molecules (93), allowing for excellent detection of non-hemorrhagic lesions following axonal shearing. Lesions on DWI seem to correlate with initial glasgow coma scale (GCS) score and coma duration in DAI (91), and are associated with poor outcome in pediatric TBI (94). Specifically, the DWI lesion load in the corpus callosum may be of particular importance (85). However, similar to the FLAIR sequence, timing of the MRI scan is imperative when assessing DWI images (95) with the number of lesions being significantly reduced at 3 months post-TBI (84).

MRI can be difficult to obtain, particularly in the critically ill, since patients’ transfer to the MRI facility may be prevented by clinical instability from intracranial and/or systemic causes. In situations where the time for MRI must for clinical reasons be extended beyond the acute phase, hemorrhagic lesions depicted in particular on SWI sequence seem to have higher prognostic value than other MR sequences. It is notable that conventional MRI sequences may still be insensitive to microstructural damage to axons and injury to white matter tracts of clinical significance can still be missed with this imaging modality (96).

### Diffusion Tensor Imaging

By using image acquisition in multiple directions, the anisotropic diffusion of water molecules can be used to create DTI, providing anatomical reconstruction images of white matter tracts and quantitative measurements of axonal injury (97). DTI is more sensitive for DAI than conventional MRI, and can be used to visualize ultrastructural changes. By adding post-processing techniques to the DTI data, diffusion tensor tractography can visualize the three-dimensional anatomy of white matter tracts (98, 99). Reduction of fractional anisotropy (FA) and increased diffusivity are observed following DAI in numerous studies (100–102), they correlate to TBI severity (103, 104) and are strongly associated to cognitive and behavioral deficits in both adult and pediatric patients (10, 105–110). In addition, DTI detection of axonal injury has been cross-validated using microdialysis (MD), where FA reduction correlated to interstitial fluid (ISF) tau levels (111). As for monitoring of axonal integrity, DTI parameters have shown signs of ongoing microstructural changes long after the acute phase (105, 107, 112–114). Longitudinal studies suggest continuous changes of DTI parameters, where FA decreases over time while diffusivity increases following DAI (102, 112–114). Measurable deterioration of white matter integrity continues beyond 24 months post-injury (105, 112–115), but may stabilize thereafter (105, 115).

In summary, DTI is a robust tool to visualize posttraumatic white matter abnormalities. However, variations in data acquisition, analysis techniques, spatial location of investigated structures, lack of correlation with clinical findings, and costs (116) still impede generalized conclusions of its applied utility in DAI. Moreover, DAI lesions seen on DTI are currently used predominantly for diagnostic purposes since patient management remains predominantly symptomatic awaiting the implementation of novel pharmacological treatments.

### Magnetic Resonance Spectroscopy

Magnetic resonance spectroscopy takes advantage of the chemical shift, a phenomenon caused by variations of proton resonance due to the local chemical environment. Although MRS has a low spatial resolution in comparison to MRI, it provides a mean of detecting and quantifying neurochemical alterations (117). *N*-acetyl aspartate (NAA), a marker for neuronal and axonal integrity found in high concentrations in neurons (118), and choline (Cho), which is increased after damage to cell membranes (119), are well-studied metabolites in relation to TBI. In most studies, decreased NAA and increased Cho is observed and in mild TBI, the NAA and Cho levels can appreciate axonal injury undetected by conventional MRI (120–124). These MRS findings are associated with neurocognitive deficits (125–127) and global outcomes (120, 121, 128). Longitudinal studies suggest recovery of decreased NAA levels in patients with mild DAI and/or better outcomes, implying marginal dysfunction of neurons and restoration of function over time (120, 122). However, complete recovery of NAA levels may also be possible following severe DAI (129).

Thus, MRS provides a mean for detecting and monitoring alterations in brain chemistry following DAI, although its clinical utility in DAI patients has still not been well defined. This is likely due to the lack of standardized protocols for measurements and interpretation of metabolite concentrations, a shortcoming that needs to be addressed in future studies.

### Neuromolecular Imaging

#### Single-Photon Emission Computed Tomography

Single-photon emission computed tomography (SPECT) uses radiopharmaceutical agents to produce images of physiologic or pathological processes. For TBI, [^99m^Tc] Hexamethylpropylenamine oxime (HMPAO) and [^99m^Tc] Ethylcisteinate dimer are the most widely used agents for evaluating regional cerebral blood flow (rCBF) and indirectly, regional cerebral metabolism (130). Following DAI, decreased rCBF commonly involving the cingulate gyrus revealed signs of frontal lobe dysfunction, despite the absence of distinct anatomical abnormalities (131, 132). One plausible explanation to these alterations is deafferentation of interconnecting white matter due to widespread axonal damage, causing reduction of metabolic activity and eventually neurocognitive deficits (133, 134).

Although SPECT cannot be independently used in the evaluation of DAI due to limited image resolution and sensitivity, it provides an available and affordable adjunct measure to other anatomical imaging modalities of white matter injury.

#### Positron Emission Tomography (PET)

Imaging of physiologic and biochemical processes following DAI is also possible by PET, using radiopharmaceutical agents labeled with positron-emitting radioisotopes such as fluorine-18 [^18^F], carbon-11 [^11^C], and oxygen-15 [^15^O]. The [^18^F]-labeled fluorodeoxyglucose (FDG) PET is widely used in brain imaging to measure local glucose metabolism, and thus regional neuronal activity. Similarly, FDG PET studies of DAI patients have revealed regional hypometabolism in medial frontal lobe structures including the cingulate gyrus (134), findings associated with neuropsychological and cognitive symptoms (134, 135). Additionally, neuroinflammatory alterations can be studied with [^11^C] PK11195, reflecting microglial activation (136). Using this tracer, widespread neuroinflammation post-TBI was observed in subcortical structures of DAI patients (136, 137). Furthermore, amyloid binding tracers commonly used in Alzheimer’s disease have recently established PET as a method for imaging of amyloid-β (Aβ) also in TBI (138, 139). Aβ retention signals were assessed in nine TBI patients, of which four had DAI, and appeared to peak within the first week post-injury (138) and correlate with white matter damage. However, in the chronic stage, Aβ retention signals were also increased with longer time from the initial injury (139).

Future studies will provide knowledge on the topographical distribution and temporal patterns of Aβ deposition following axonal injury, and their relation to the development of neurodegenerative diseases. Although PET scan is a powerful tool which offers superior image resolution, sensitivity, and quantification of regional radioactivity concentrations compared to SPECT (140), its main disadvantage remains the requirement of an on-site cyclotron which considerably increases cost and limits availability.

## Fluid and Structural Biomarkers

Per definition, biomarkers are molecules measurable in biological fluids and structures given that the measurable value is related to a biological or pathological process in the body (141, 142) (Table 1).

**Table 1:** Blood and cerebrospinal fluid (CSF) levels of common axonal injury biomarkers (neurofilament, tau, SBDP and amyloid-β) in clinical TBI.

Reference	Biomarker	*N*	Type of injury	Compartment	Biomarker levels—control group	Biomarker levels—TBI	Major findings
Zurek et al. (159)	pNF-H	49	Pediatric severe TBI (DAI *n* = 9)	Blood	N/A	TBI: 12 (12–1,482) pg/ml DAI: 159 (12–867) pg/ml	Increased levels in DAI

Al Nimer et al. (167)	NF-L	182	Mild: *n* = 15	Blood, CSF	Serum: 7.9 ng/l	Serum: 400 (181–865) ng/l	Serum NF-L correlated negatively to outcome in all TBI patients. No predictive value of NF-L on outcome in DAI patients
			Moderate: *n* = 39		CSF: 138 ± 31 ng/l	CSF: 7,026 (2,610–19,204) ng/l	
			Severe: *n* = 128 (DAI: *n* = 40)				

Ljungqvist et al. (169)	NF-L	9	DAI	Blood	10.8 ± 5.4 pg/ml	347.12 ± 220.65 pg/ml	30-fold increase of NF-L in DAI. NF-L levels were related to DTI parameters

Zetterberg et al. (163)	NF-L	14	Amateur boxers	CSF	≤125 ng/l	845 ± 1,140 ng/l	Increased in boxers, remained elevated at 3 months

Neselius et al. (164)	NF-L	30	Olympic boxers	CSF	135 ± 51 ng/l	532 ± 553 ng/l	Increased in >80% of boxers

Shahim et al. (165)	NF-L	31	Professional ice hockey players	CSF	238 (128–526) pg/ml	410 (230–1,440) pg/ml	Increased levels in players with PCS more than 1 year

Shahim et al. (168)	NF-L	72	Severe TBI (DAI: *n* = 33)	CSF, blood	In CSF not specified	In CSF not specified	Increased serum levels in TBI and predicted poor outcome. Similar dynamics in blood and CSF
					Blood: 13 (11–17) pg/ml	Blood (GCS 6–8): 196 (89–413) pg/ml; (GCS 3–5): 107 (67–190) pg/ml	

Shahim et al. (162)	NF-L	49	Amateur boxers (*n* = 14)	Blood	9 pg/ml (IQR 7–14)	Boxers: 22 pg/ml (IQR 18–34)	Marked increase in boxers 7–10 days after bout. Highest levels in hockey players at 144 h post-concussion
			Professional hockey players (*n* = 35)			Hockey players: Elevated values compared to controls^b^	

Bagnato et al. (166)	NF-L	10	Severe, persisting DOC following severe TBI	CSF	1,173 pg/ml (670–3,643)	4,458 ng/ml (695–23,000)	Very high levels of NF-L compared to controls suggesting possible ongoing axonal degeneration up to 19 months following severe TBI

Bazarian et al. (182)	c-tau	35	Mild TBI	Blood	N/A	4.85 ± 9.23 ng/ml	C-tau unreliable as a predictor of 3-month outcome

Bulut et al. (177)	t-tau	60	Mild TBI	Blood	86 ± 48 pg/ml	188 ± 210 pg/ml	Levels in high-risk patients (GCS score 14.3 ± 0.73) were significantly higher than in low-risk patients (14.9 ± 0.33)

Shahim et al. (176)	t-tau	28	Concussed professional ice hockey players	Blood	Pre-season: 4.5 pg/ml (0.06–22.7)	Post-concussion: 10.0 pg/ml (2–102)	Peak t-tau immediately post-concussion

Shahim et al. (172)	tau-A, tau-C	28	Concussed professional ice hockey players	Blood	Values are given in graphs (no average)	Values are given in graphs (no average)	No significant increase in tau-A levels but elevated tau-C levels post-concussion compared to pre-season. Tau-A levels correlated with the duration of post-concussive symptoms.

Franz et al. (207)	t-tau	29	Severe TBI (DAI: *n* = 7)	CSF (lumbar, ventricular)	193 pg/ml (16–326), 109 pg/ml (69–159)	1,756 pg/ml (35–5,720)	Increased tau levels early post-TBI; peak in second week

Zetterberg et al. (163)	t-tau, p-tau	14	Amateur boxers	CSF	t-tau: 325 ± 97.7 ng/l	t-tau: 449 ± 176 ng/l	Increased levels of t-tau in boxers after a bout mainly in those who received many or high-impact hits, resolved at 3 months
					p-tau: 46.4 ± 14.5 ng/l	p-tau: 37.9 ± 10.2 ng/l	

Neselius et al. (164)	t-tau, p-tau	30	Olympic boxers	CSF	t-tau: 45 ± 17 ng/l	t-tau: 58 ± 25 ng/l	Increased levels of t-tau in >80% of boxers. Increasing levels during first 6 days, resolved after 14 days
					p-tau: 23 ± 6 ng/l	p-tau: 21 ± 7 ng/l	

Oliver et al. (178)	t-tau	19	American football players	Blood	t-tau: 3.7 ± 0.9 pg/ml	t-tau: 3.0 ± 1.2 pg/ml	No difference between players and non-contact swim athletes following a season

Pineda et al. (193)	SBDP	41	Severe TBI (Diffuse TBI/DAI: *n* = 23)	CSF	Arbitrary units	Arbitrary units	SBDP150 elevated up to 24 h, SBDP145 up to 72 h, SBDP after 24 h post-injury

Brophy et al. (194)	SBDP	38	Severe TBI (DAI: *n* = 20)	CSF	Arbitrary units	Arbitrary units	SBDP150 and SBDP145 elevated 24–72 h post-injury, SBDP120 elevated 24–120 h post-injury

Mondello et al. (185)	SBDP	40	Severe TBI (DAI: *n* = 14)	CSF	SBDP145: 0.52 ± 0.22 ng/ml^a^	SBDP145:14.42 ± 0.91 ng/ml	Higher SBDP145 and SBDP120 in TBI patients, particularly in patients who died
					SBDP120: 1.21 ± 0.48 ng/ml^a^	SBDP120: 6.05 ± 0.28 ng/ml	

Siman et al. (190)	SNTF	17	Mild TBI	Blood	Arbitrary units	Arbitrary units	Associated with DAI, as evaluated by DTI, and cognitive impairment at 3 months

Siman et al. (191)	SNTF	28	Professional ice hockey players	Blood	Arbitrary units	Arbitrary units	Elevated levels correlated with concussion and delayed return to play

Raby et al. (206)	Aβ40, Aβ42	6	Severe DAI	CSF	Aβ40: 1.59 ± 0.53 ng/mg	Aβ40: 0.94 ± 0.08 ng/mg	Aβ42 increased in CSF by TBI compared to controls, peaked in week 1, declined over next 2 weeks
					Aβ42: 0.38 ± 0.2 ng/mg	Aβ42 1.17 ± 0.11 ng/mg	

Franz et al. (207)	Aβ42	29	Severe TBI (DAI: *n* = 7)	CSF [lumbar (*n* = 14), ventricular (*n* = 15)]	DM: 284 pg/ml (172–564)	167 pg/ml (120–477)	Low CSF levels associated with a poor outcome
					HD: 388 pg/ml (256–768)		

Zetterberg et al. (163)	Aβ40, Aβ42	14	Amateur boxers	CSF	Aβ40: 19,400 ± 5,050 ng/l	Aβ40: 19,300 ± 2,740 ng/l	Aβ levels not significantly altered
					Aβ42: 773 ± 114 ng/l	Aβ42:858 ± 128 ng/l	

Olsson et al. (204)	Aβ42	28	Severe DAI	CSF, blood	N/A	CSF: peak 129 (60–171) pg/ml (d5–6)	Levels increased stepwise, peak day 5–6
						Plasma: peak 57 (37–68) pg/ml (d5–6)	

Mondello et al. (205)	Aβ42	12	Severe TBI (DAI: *n* = 6)	CSF, blood	CSF: 537.6 pg/ml (350.8–710)	CSF: 105.9 pg/ml (46.0–216.2)	Decreased in CSF and increased in plasma post-TBI
					Plasma: 7.3 pg/ml (6.1–8.7)	Plasma: 17.0 pg/ml (14.7–28.6)	

Shahim et al. (165)	Aβ42	31	Professional ice hockey players	CSF	1,094 (845–1,305) pg/ml	1,000 (757–1,040) pg/ml	Lower levels in PCS

Shahim et al. (210)	Aβ40, Aβ42	28	Professional athletes	CSF	Exact values not reported	Exact values not reported	Lower values in athletes with repeated concussions

*Articles including patients with diffuse axonal injury (DAI) and mild TBI where axonal biomarkers were measured are presented. Data are given as mean ± SD, median and range as appropriate*.

*DM, patients with dementia; DOC, disorder of consciousness; HD, patients with headache; ICP, intracranial pressure; IQR, Interquartile range; NF-L, neurofilament- Light; PCS, post-concussion syndrome; pNF-H, phosphorylated neurofilament-heavy; SBDP, spectrin breakdown products; SNTF, spectrin N-terminal fragment; TBI, traumatic brain injury; UCH-L1, ubiquitin carboxy-terminal hydrolase L1; DTI, diffusion tensor imaging*.

*^a^145 and 150 kDa αII-spectrin breakdown products*.

Biomarkers may be subdivided into four categories: diagnostic, prognostic, predictive, and pharmacodynamic and can potentially be used to examine injury severity, monitor pathophysiology of injury, explain adaptive and recovery processes, guide management, predict response to treatment and estimate prognosis following DAI/TBI (141–143). Biomarkers can thus be considered a reflection of the mechanisms resulting in axonal injury, where the underlying structural changes are to a large extent related to the activation of the calpain and caspase enzymes. These enzymes belong to the cysteine protease family and play an important role in cell necrosis and apoptosis (144–150). They are activated by calcium influx leading to cytoskeletal disruption including an impaired axoplasmic transport, axonal swelling and eventually axonal transection/lysis (35, 151–154).

Thus, following TBI and in particular following axonal injury, a delayed axonal transection may occur resulting in the release and accumulation of various biomarkers which can be detected in plasma, CSF, and ISF using cerebral microdialysis (MD) (141, 155) and can, therefore, be used for monitoring. Biomarkers reviewed more extensively below are neurofilaments, tau, Spectrin breakdown products (SBDP) and Aβ and are summarized in Tables 1 and 2.

**Table 2:** Results from cerebral microdialysis (MD) studies of commonly used biomarkers for monitoring axonal injury in clinical DAI.

Reference	Biomarker	*N*	Type of injury	Biomarker levels—control group	Biomarker levels—TBI	Major findings
Magnoni et al. (218)	NF-L	16	Severe TBI^a^	104 pg/ml [0–1,201 (seemingly normal cortex)]	1,555 pg/ml [range 1,152–2,012 (pericontusional)]	Higher levels in focal injury and pericontusional areas than in DAI
Marklund et al. (217)	t-tau	8	Severe TBI^b^	No controls available. Level of detection 75 pg/ml	2,881 ± 1,774 pg/ml (121–6,500)	Higher levels in focal/mixed TBI than in DAI
Magnoni et al. (218)	t-tau	16	Severe TBI^a^	3,469 pg/ml [1,684–8,691 (*n* seemingly normal cortex)]	15,950 pg/ml [11,390–27,240 (pericontusional)]	Higher values in focal injury/pericontusional than in DAI
Magnoni et al. (111)	t-tau	15	Severe TBI^c^	32 pg/ml (detection level)	12,813 pg/ml (4,858–18,744) first 24 h	High initial t-tau levels declined over time, correlated with DTI
Marklund et al. (217)	Aβ42	8	Severe TBI^b^	15.6 pg/ml (detection level)	167 pg/ml (31–295)	Higher levels of Aβ42 in DAI compared to focal/mixed TBI patients
Magnoni et al. (218)	Aβ1-x	16	Severe TBI^a^	1,023 pg/ml [778–1,968 (seemingly normal cortex)]	270 pg/ml [83–417(pericontusional)]	Lower Aβ levels in focal injury/pericontusional than in DAI
Magnoni et al. (111)	Aβ1-x	15	Severe TBI^c^	4.9 and 7.81 pg/ml (detection level)	756 pg/ml (575–1,079) first 24 h	Low initial Aβ levels that rose over time
Helmy et al. (219)	42 cytokines	12	Severe DAI	N/A	N/A	Cerebral production of numerous cytokines, of which 16 peaked at defined time points post-injury^d^, was detected
Helmy et al. (222)	42 cytokines	20	Severe DAI	N/A	N/A	Treatment with rhIL1ra influences microglial phenotype as evaluated by MD cytokines

*Only MD studies where data is available for DAI patients are included*.

*Aβ, Amyloid-β; DAI, diffuse axonal injury; DTI, diffusion tensor imaging; IL, interleukin; N/A, non applicable; NF-L, neurofilament light; rhIL1ra, recombinant human interleukin-1 receptor antagonist; TBI, traumatic brain injury*.

*^a^Nine were classified as DAI according to Marshall CT classification*.

*^b^Three patients had DAI*.

*^c^Most patients (11/15) had DAI according to Marshall CT classification. No MD catheters were placed in pericontusional areas*.

*^d^These cytokines included IL10, IL12p40, IL12p70, IP10, monocyte chemotactic protein-1, monocyte chemoattractant protein 3 (MCP3), monocyte inflammatory protein 1a (MIP1a), MIP1b, platelet derived growth factor AA (PDGF-AA), transforming growth factor-a (TGF-a) and vascular endothelial growth factor (VEGF)*.

### NFL

Neurofilaments (NF) are important components of the axonal cytoskeleton, mainly involved in synapses and neurotransmission (156). They represent intermediate neuronal filaments and include three major subunits: neurofilament light (NF-L), neurofilament medium and neurofilament heavy chain (NF-H) (156). The latter becomes phosphorylated (pNF-H), likely by TBI-induced calcium influx, which can alter axonal integrity (156). Of the three subunits, NF-L is rapidly degraded following axonal injury (157) making it a rather sensitive and specific biomarker for the detection of injured axons (5, 141, 158). Following axotomy, phosphorylated neurofilaments (pNF-H) are released in CSF and blood, correlating with injury severity and outcome both in the pediatric and adult population (159, 160).

Neurofilament light fragments can also be identified in both blood and CSF in TBI (143, 161, 162). Following mild and repetitive impacts to the head like those occurring in contact sports such as boxing, American football and ice hockey, increased levels of NF-L may be associated primarily with injury to long, myelinated axons (8, 162–165). Recently, very high levels of NF-L compared to controls were found in 10 patients with impaired level of consciousness following TBI, with the samples taken at least 10 months following injury, results suggesting ongoing axonal degeneration (166).

Increased serum and CSF NF-L levels in TBI patients did also correlate with clinical outcome although without any predictive value for DAI (167). In another study that included 72 patients with severe TBI (of which 33 had DAI), initial NF-L levels independently predicted clinical outcome (168). Additionally, in patients with severe DAI, a 30-fold increase in serum NF-L was recently found (169).

Repeated biomarker sampling during the course of the disease as well as the correlation with advanced neuroimaging is expected to better discern the role of neurofilaments in DAI, their contribution in the pathophysiology of DAI and their prognostic value on outcome.

### Tau

Tau is a structural protein with six isoforms in humans and is a normal constituent of axons. Four distinct isoforms of tau are usually applied in biomarker studies; total-tau (t-tau), cleaved microtubule-associated tau (c-tau), phosphorylated tau (p-tau), and the recently discovered tau-A (155, 170–172). Tau has been linked to axonal damage following TBI (141, 173). Specifically, the presence of c-tau in CSF is a highly sensitive indicator of axonal injury (35).

In patients with DAI, t-tau and p-tau levels also increase rapidly within hours after injury, especially in CSF (35, 170, 174). Increased CSF levels of t-tau were found in boxers after repetitive head injury, although this increase was modest compared to that of NF-L (163, 164).

The Simoa platform has shown excellent analytical sensitivity for tau in serum (8, 175). Serum c-tau levels are increased but at much lower levels than in CSF and have been used as an indicator of blood–brain barrier damage (35). Compared to off-season levels, serum t-tau levels were elevated in ice hockey players sustaining a concussion, with the highest levels detected immediately after injury (141, 176). In mild TBI, t-tau levels were found to be higher in high-risk patients with greater likelihood for TBI-related complications than in low-risk individuals (177). However, no difference in serum tau levels was recently noted in American football athletes (178).

In DAI, CSF c-tau correlated negatively with the degree of clinical improvement (170, 179, 180). Furthermore, increased serum c-tau levels were associated with poor outcome in patients with mild TBI (181). In contrast, another study found that c-tau is not a reliable predictor for 3-month outcome following mild TBI (182). In concussed professional ice hockey players, the levels of the newly discovered biomarker tau-A correlated with the duration of symptoms post-injury, and may possibly predict return to play (172).

The association of elevated tau levels with axonal damage is well established. Especially in severe DAI, high tau levels are associated with worse outcome. Ongoing and future research efforts need to focus more on its possible correlation with the extent of injury, interaction with other blood and CSF biomarkers, long-term sequelae, and clinical outcome.

### Spectrin Breakdown Products

Spectrin is a cytoskeletal protein playing an important role in the cytoskeletal structure and maintenance of plasma membrane (183). In DAI, spectrin is proteolytically cleaved by calpain, resulting in cytoskeletal destruction (184). SBDP are increased in human CSF and blood following severe TBI and may predict injury severity and outcome (5, 185).

In rodents, SBDP are detected within minutes after DAI (186–188). In human TBI, αII-spectrin N-terminal fragment (SNTF) accumulates in injured axons, rises in serum as early as 1 h after mild TBI and correlates with cognitive impairment (141, 189–191). Importantly, SNTF immunoreactive axons have been also identified both in mild and severe TBI (192). Serum SNFT levels were also increased early after concussion in ice hockey players, particularly in more severe injuries (141, 191).

In severe TBI patients, elevated levels of calpain-mediated 150- and 145-kDa SBDP in CSF were found 24–72 h post-injury (193, 194) which were associated with the initial injury severity and 6-month outcome (193). In addition, CSF calpain-mediated SBDP levels correlated positively with the severity of injury, lesion size, and behavioral deficits in severe TBI, suggesting that CSF SBDPs could be used to evaluate the magnitude of axonal injury and predict functional deficits (193).

Spectrin breakdown products are relatively new biomarkers detected in serum and CSF. Therefore, ample evidence on their significance in the clinical setting is lacking. However, available data suggests that they represent promising molecules in determining the extent of axonal injury and its association to outcome.

### Amyloid-β Peptides

Axonal injury in TBI has been characterized by amyloid precursor protein (APP) immunohistochemistry, accumulating at sites of axonal transport failure (8, 195, 196). The presence of APP-positive axonal bulbs and grossly swollen axons are main findings in DAI (195), observed within hours in severe TBI patients (197). However, APP is not a specific diagnostic marker of DAI, since it may also be detected in non-traumatic, ischemic, axonal injury and in multiple sclerosis plaques (35, 198–200). APP co-accumulates with the enzymes necessary for its cleavage to Aβ peptides, such as presenelin-1 and beta-site APP-cleaving enzyme (19, 201, 202). Conversely, notable amount of Aβ has been repeatedly found in axonal bulbs (17, 32, 201–203).

By cleaving APP, the Aβ peptides Aβ40 and Aβ42, the substrates for Aβ aggregates/plaques also observed in Alzheimer’s disease, are produced (19). APP and Aβ species are rapidly detectable following TBI in plasma (204, 205), CSF (205–207) and ISF (208, 209). In severe TBI, monomeric Aβ levels in ventricular CSF were increased stepwise until 5–6 days after injury, although not in plasma (204). Conversely, a more recent study using an ultrasensitive digital immunoassay evaluating 12 severe TBI patients of which 6 had DAI, reduced CSF levels of Aβ42 direct after injury with lower levels in patients who died 6 months post-injury were observed. In the same study, plasma levels were increased with lower levels detected in surviving patients (205). The differences in analytical methods may partly explain the discrepancy in the results between these studies. Additionally, the latter study also included patients with focal TBI, although no difference in Aβ levels was observed between TBI subtypes (205). Similarly, lower CSF levels of Aβ40 and Aβ42 were recently detected in professional athletes following concussions (165, 210).

An increased interest in soluble intermediary Aβ oligomers/protofibrils as the pathogenic form of Aβ has emerged since they are likely to contribute to the development of Alzheimer’s disease (211, 212). Aβ oligomers have been detected in lumbar CSF from severe TBI patients, were elevated in patients with poor neurological outcomes and were negatively correlated to CSF Aβ42 (213). Therefore, it is plausible that aggregation of Aβ into oligomers may explain the reduced levels of CSF Aβ seen in TBI. However, the corresponding brain tissue levels of soluble intermediary Aβ species and their role in human DAI remains to be established. In addition, these potentially neurotoxic species could represent a pathophysiologic link between DAI and Alzheimer disease-like dementia.

The association between TBI and the development of neurodegenerative diseases, in particular Alzheimer’s disease, has been repeatedly demonstrated (24, 214). Longitudinal monitoring of Aβ dynamics may provide further knowledge of neurodegenerative processes following DAI. Aβ42 levels, which can be monitored in both CSF and blood are likely the most promising biomarker from the amyloid family for detecting the extent and severity of DAI. In addition, specific monitoring of potentially neurotoxic oligomeric and protofibrillar Aβ species will become possible using newly developed antibody-based PET imaging (215). This will further increase the understanding of the potential link between DAI and neurodegeneration.

### Biomarkers and Cerebral Microdialysis

Cerebral MD is a neurocritical care monitoring technique predominantly used in patients with severe TBI and subarachnoid hemorrhage (Table 2). Its main advantage is that it allows continuous neurochemical monitoring of factors located in the extracellular, interstitial fluid (216).

Using MD on 8 severe TBI patients, particularly high ISF Aβ42 values were found in the three DAI patients (217). In another MD study, in nine DAI patients the initial Aβ levels inversely correlated with tau levels in ISF (218), suggesting that low Aβ levels in regions with elevated tau may be due to reduced synaptic activity after axonal injury (218).

Tau was also evaluated by MD in eight severe TBI patients (217). Although mean t-tau levels were clearly above the detection limit in the first days after injury, patients with focal/mixed injury (*n* = 5) had lower levels compared to those with DAI (*n* = 3). Conversely, in a previous MD study, higher tau values were observed pericontusionally in focal TBI patients when compared to tau levels obtained from DAI patients with the MD catheter placed in structurally normal frontal cortex. Early tau levels were inversely correlated with the initial Aβ levels (155, 218). In this study, NF-L levels were also higher in pericontusional tissue (218). Further, in a study of 15 patients with severe TBI (11/15 had DAI), initially high t-tau levels in ISF declined over time and a correlation with DTI and reduced brain white matter integrity in the region of MD sampling was observed, suggesting that increased tau levels reflected axonal injury (111).

The cytokine response was evaluated by MD in patients with severe DAI suggesting that cytokine production is highly compartmentalized with significant differences between brain parenchymal and systemic concentrations (219–222). Several cytokines are produced in different phases of the inflammatory response (220). It has been also shown that in DAI patients, treatment with an interleukin receptor-1 antagonist increased microglial activation, altering the cytokine profile to one consistent with an M1 microglial phenotype, providing proof of concept that an anti-inflammatory treatment administered systemically can alter cerebral cytokine productions in human TBI (222). These data suggested that the patterns of cytokine release in ISF are promising targets for biomarker research in DAI.

Following DAI, markers analyzed in MD samples indicating acute and chronic neuroinflammation may potentially be used to guide treatment, as measures for pharmacological response, and/or for tissue outcome. Specifically, MD may aid in detecting factors related to the progression of the disease and in the understanding of the pathophysiology of axonal injury. Therefore, it is likely that data from MD, in combination with widely used measures such as ICP-CPP guided monitoring and protocols, will contribute to the understanding of the pathophysiology of DAI and potentially aid in evaluating novel pharmacological treatments.

However, MD is time consuming, usually used for low-molecular weight molecules and frequently characterized by high variability and lack of standardization. In addition, MD remains a predominantly focal measurement technique. To date, there is insufficient data arguing for MD to be used as a clinical decision tool for DAI patients and should rather be considered an integral part of the multimodality monitoring during neurocritical care as well as a research tool.

### Other Biomarkers

There are numerous additional biomarkers associated to CNS injury that can potentially be related to axonal injury. Examples of such biomarkers are Glial Fibrillary Acidic Protein, ubiquitin carboxy-terminal hydrolase L1 myelin basic protein, microtubule-associated protein 2, protein S-100B and neuron-specific enolase, among others (5, 35, 141–143, 223–228). Especially the astrocytic protein S-100B is a promising biomarker across all injury severities, with higher S-100B levels observed in focal compared to diffuse TBI (229, 230). Moreover, it has been shown that S-100B correlates with Marshall CT classification scores (231). Furthermore, following TBI, neuroinflammation may as previously noted play an important role as a key secondary injury factor (141, 232). Specifically, it has been repeatedly shown both in the experimental and clinical setting that cytokines such as Tumor Necrosis Factor-α and Interleukins (ILs) 1β, 6, 8, and 10 are increased following TBI both in blood and CSF (233).

Since the above mentioned biomarkers are not specific for axonal injury, at present, their elevations in CSF or serum should be interpreted with caution from both the diagnostic and predictive perspective with regards to DAI.

### Limitations of Biomarkers

Although considerable progress has been made in the recent years in research, the quest for TBI-specific biomarkers continues. The currently used biomarkers commonly have different specificity- and/or sensitivity, limited availability for bedside analysis and use in daily practice as well as variable half-lives. Moreover, some can also be released from other organ systems during different disease or injury processes.

Biomarkers obtained from CSF and ISF are theoretically considered better and more reliable sources compared to blood biomarkers. Therefore, it is preferable to obtain samples from these compartments whenever possible. However, in particular in mild and moderate TBI, there is generally no clinical indication for CSF and, for obvious reasons, ISF sampling by invasive means. On the other hand, blood samples are easily accessible in almost every TBI patient.

Nevertheless, important issues relevant to TBI-related blood biomarkers include their relatively low concentrations, proteolytic degradation, the requirement of carrier proteins and the different permeability across the blood–brain barrier for certain biomarkers (141).

Standardization and validation of biomarker levels are other important issues since different methods of analysis, preparation and sample quality can provide different results among laboratories and centers which can cause problems during the interpretation and comparison of results (234). As the field of biomarker research is expanding, CNS sensitivity and/or specificity increases their importance. Analysis of many currently used biomarkers requires specialized research laboratories which are not available on a daily basis. Additionally, difficulties may be encountered when attempting to assess biomarker half-time, especially in those that are continuously released from the brain following injury and in those with complex elimination or degradation mechanisms (230).

## Recommendations for Monitoring Axonal Injury

1. ICP and CPP monitoring as well as ICP-CPP guided therapy are advised in all severe TBI patients with suspected axonal injury and decreased level of consciousness especially in the initial post-injury phase.
2. Conventional MRI scan sequences such as FLAIR, DWI, and SWI should be considered in the first post-injury period following TBI to detect and confirm the presence of DAI.
3. Advanced MRI techniques such as DTI and MRS are useful modalities for further delineation of axonal damage in TBI, particularly in the subacute and chronic phase.
4. Due to high cost and limited availability, PET scanning is recommended solely as a valuable research tool although not to date in clinical management.
5. Biomarkers specific for axonal injury can be analyzed in blood and CSF from the acute to chronic post-injury period in TBI, aiming to aid in the understanding of the axonal injury process, follow the course of the disease, monitor for possible deterioration, estimate the extent of axonal injury and aid in prognostication.
6. Repeated clinical examinations and neuropsychological tests can provide invaluable information on the extent of injury, prognosis and for monitoring possible recovery or exacerbation of cognitive functions and mental status.

## Conclusion

Although axonal injury has traditionally been associated with an impaired level of consciousness and poor prognosis, patients with confirmed axonal damage can achieve a good clinical outcome. Using advanced neuroimaging, axonal injury is increasingly recognized also in mild TBI or sports-related concussions. Many tenets of the pathophysiology of axonal injury are being elucidated through major efforts in basic science and medical research. Nonetheless, it remains an exceedingly complex subtype of TBI with many unknown secondary pathological processes. Since the secondary injury cascades are continuing for a considerable time post-injury, monitoring is critically important for clinical as well as research purposes. Advanced imaging techniques such as MRS, DTI and PET show promise in better identifying and quantifying axonal injury and its importance for patient outcome. In addition, both invasive and non-invasive neurocritical care techniques are becoming increasingly important in monitoring axonal injury. Numerous biomarkers with, plausibly, high specificity for axonal damage have been and are being developed. This evolving field of TBI research is promising for the development of bedside, rapid analysis kits for small-volume body fluids. When these novel biomarkers are available for routine use as monitoring tools for axonal injury, they may in the future aid in the detection and prevention of secondary axotomy and atrophy of white matter tracts. They may also be used as secondary outcome measures in DAI, assist in the development of novel therapies, guide treatment, and monitor treatment response. Finally, the short-and long-term monitoring options for axonal pathology and its progression described in this review may become crucial for the prevention of neurodegeneration at the chronic stage in DAI.

## Author Contributions

Study concept and design, drafting of the manuscript, analysis and interpretation of data, and critical revision of the manuscript: PT, SAH, and NM. Acquisition of data: PT and SAH. Study supervision: NM.

## Conflict of Interest Statement

The authors declare that the research was conducted in the absence of any commercial or financial relationships that could be construed as a potential conflict of interest.
